# Whole Exome Sequencing reveals a NLRP3 mutation in exon 5 in a patient with CINCA

**DOI:** 10.1186/1546-0096-13-S1-P45

**Published:** 2015-09-28

**Authors:** S Melo Gomes, J Arostegui, E Omoyinmi, A Standing, N Klein, H Lachmann, P Hawkins, P Brogan

**Affiliations:** 1 Institute of Child Health, Rheumatology, London, UK; 2 Hospital Clinic, Immunology, Barcelona, Spain; 3 Royal Free Hospital, NAC, London, UK

## Introduction

Cryopyrin-associated periodic Syndromes (CAPS) are caused by heterozygous mutations in the NLRP3 gene. More than 80 disease causing mutations have been identified, mostly clustered in NLRP3 exon 3, but also described in exons 4 and 6. However, up to 50% of clinically diagnosed CAPS patients (with identical clinical features and response to anti-IL-1 treatment) show no mutation in NLRP3 detected by conventional DNA sequencing analysis of exons 3, 4 and 6. Recently somatic NLRP3 mosaicism has been shown to account for up to 70% of these mutation negative CAPS patients.

## Objective

To ascertain a genetic cause in a patient with a CINCA phenotype labeled as NLRP3 mutation negative.

## Methods

Massively Parallel Sequencing (MPS) and Whole Exome Sequencing (WES) were performed on DNA extracted from peripheral blood. WES results were confirmed by conventional Sanger sequencing.

## Case report

Twenty month old boy who presented with an urticarial-like rash since birth. At 6 months of age he was noted to have frontal bossing with increasing head circumference, hepatosplenomegaly, and papilledema with no signs of uveitis. A mild conductive hearing impairment was detected, but no sensorineural hearing loss. Inflammatory markers were raised with a CRP of 40mg/L.

Brain MRI showed signs of hydrocephalus and a ventriculo-peritoneal shun was inserted.

A clinical diagnosis of CAPS was made at this point. Sanger sequencing of NLRP3 exons 3, 4 and 6 was consistent with wild-type.

Over the following months his clinical status deteriorated with slowing development and significant failure to thrive with growth below the 0.4^th^ centile. Inflammatory marks were persistently raised.

Anakinra treatment was started at 12 months of age, resulting in a marked clinical and serological improvement, with normalization of CAPS disease activity score and inflammatory response at a dose of 3mg/kg/day.

MPS ofNLRP3 exons 3, 4 and 6 didn't reveal any somatic mutations. WES was then performed for the proband and his parents revealing a potentially damaging heterozygous variant in NLRP3 exon 5 (c.G2336T; p.G779V), which segregated with disease. Sanger sequencing of exon 5 confirmed these findings.

## Conclusion

To the best of our knowledge this is the first potentially disease associated mutation in NLRP3 exon 5. Although we have not yet performed functional studies, in silico prediction is consistent with a damaging role.

Our work shows that other NLRP3 exons apart from 3, 4 and 6 should be screened for germline and/or somatic mutations in patients with a clinical diagnosis of CAPS.

## Consent to publish

Written informated consent for publication of their clinical details was obtained from the patient/parent/guardian/relative of the patient.

